# Carpopedal spasm in a 4 year old boy with persisted vomiting and dyselectrolytemia in Wesley Guild Hospital Ilesa

**DOI:** 10.11604/pamj.2019.33.43.17770

**Published:** 2019-05-21

**Authors:** Oluwasola Julius Oke, Kazeem Olarenwaju Amoo, Ifeoluwa Alex Akinwumi, Omomayowa Omotola Fawale, Adewuyi Temidayo Adeniyi, Emmanuel Oluwatosin Adeniji, Hammed Hassan Adetola, Busayo Gideon Ologun, Oluwatobi Faith Folarin, Dedmilade Kehinde Kuti

**Affiliations:** 1 Department of Paediatrics and Child Health, Obafemi Awolowo University, Ile Ife, Nigeria; 2 Department of Paediatrics, Wesley Guild Hospital, Obafemi Awolowo University, Ilesa, Nigeria

**Keywords:** Child, carpopedal spasm, dyselectrolytemia

## Abstract

Carpopedal spasm have various causes ranging from dsyselecrolytemia, syndromic, metabolic or endocrine causes. Any of these could cause a decrease in ionized calcium and tetany. Excessive vomiting leading to alkalosis, hypokaleamia and decreased ionised calcium should be kept in mind for early etiological diagnosis of carpopedal spasm. We report a case of 4-year-old boy presenting with a history of recurrent painful spasm and flexion of bilateral hands following excessive vomiting and electrolyte derangement.

## Introduction

Carpopedalspasms are frequent and involuntary muscle contractions in the hands and feet with associated pain. Hypocalcemia, low calcium levels can cause carpopedal spasms as a warning sign [1]. It occurs due to nerves and muscles hyperexcitability from decreased extracellular ionized calcium. Tetany was typically realized when ionized calcium level was lower than 1.1 mmol/L or corrected total serum calcium level falls below 7.0 mg/dL [1]. Reduction in corrected total serum calcium was frequently related with reduced ionized calcium [1]. Acid-base and electrolyte disturbances may arise from excessive vomiting [2]. Metabolic alkalosis and hypokalaemia can occur due to loss of gastric acid and potassium in the vomitus [2, 3]. Alkalosis leads to dissociation of hydrogen ion from albumin. Hence, calcium can bind to albumin causing decrease in free ionised calcium [2]. Hypomagnesaemia occurs by similar mechanism and is worsened by alkalosis as a result of intracellular shift of potassium [4]. Carpopedal spasms can occur from hypocalcaemia, hypomagnesaemia, hypokalaemia or alkalosis of which, the patient had.

## Patient and observation

**Figure 1:**
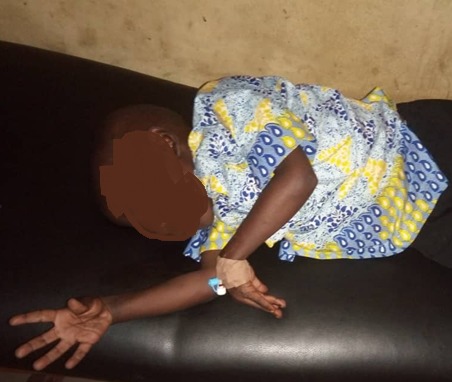
Photograph showing a 4 year old boy with carpopedal spasm secondary to dyselectrolyteamia

A 4-year-old boy was brought to the children emergency department of Wesley Guild Hospital Ilesa with painful spasm and flexion of bilateral hands (Figure 1). He had multiple episodes of vomiting for a day and 2 days history of fever before the admission. He had presented in children emergency 2 weeks earlier with similar complaint of painful spasm of bilateral hands, however with evidence of severe dehydration after several episodes of vomiting. There was no history of diarrhea, no spasm of other parts of the body and no lock jaw. There was no history of any laxative or diuretic use, no difficulty in opening the mouth or swallowing. He was then hydrated with ringers lactate and Oral Rehydration Solutions (ORS). Calcium and magnesium supplement was given and discharged home after 3days on admission. He was on calcium and magnesium supplement after discharge for about 10 days which he stopped 3days prior to his re-admission with similar complaints of painful spasm and flexion of bilateral hands. On examination, he was small for age, weight is 11kg (68.8% of expected). His body mass index was 12.2 kg/m^2^. Abdomen is flat, soft and moves with respiration. Vital signs are normal and physical examination revealed mild dehydration as evidenced by dry oral mucosa, and tender bilateral carpopedal spasm (Figure 1). Laboratory results revealed hypocalcaemia (ionised calcium 1.00 mmol/L), hypomagnesaemia (magnesium 0.4 mEq/L), hypokalaemia (potassium 2.0 mEq/L), metabolic alkalosis (serum bicarbonate 29 mEq/L), hypochloraemia (chloride 90 mEq/L), serum creatinine 44μmol/l), FBC (normal), MP 1+, urinalysis (normal), normal serum albumin 4 g/dl and spot urine sample showed (ca/creatinine ratio 0.37). He was managed with intravenous fluids (4.3% D/S), calcium and magnesium supplement, IV diazepam and antimalarial. Hypokaleamia was also corrected Carpopedal spasm and electrolyte derangement resolved after 4 days and was the discharge home. He has been coming to neurologic clinic for follow up.

## Discussion

Ionized calcium was the active form responsible for tetany [1]. Alkalosis increased calcium binding to albumin, which decreased ionized calcium. Hence, tetany might arise despite normal total serum calcium [1]. Metabolic alkalosis from recurrent vomiting in this patient could have resulted into the carpopedal spasm [2]. The patient also had hypomagnesemia, which will also cause hypokalemia and aggravate symptoms of tetany [3]. Generally, hypokalemia causes tetany in relationship with alkalosis [4]. Though, hypokalemia has similarly been reported to cause tetany in the absence of alkalosis [4].

## Conclusion

Excessive vomiting leads to alkalosis, hypokaleamia and decreased ionised calcium and carpopedal spasm. Hence, serum electrolyte including serum calcium, magnesium, potassium and bicarbonate should be done promptly in all patients with persistent vomiting from any cause to detect dyselectrolytemia early and forestall late complication such as carpopedal spasm and tetany.

## Competing interests

The authors declare no competing interests.
